# Metabolic Hormones, Apolipoproteins, Adipokines, and Cytokines in the Alveolar Lining Fluid of Healthy Adults: Compartmentalization and Physiological Correlates

**DOI:** 10.1371/journal.pone.0123344

**Published:** 2015-04-07

**Authors:** Carlos O. Mendivil, Henry Koziel, Joseph D. Brain

**Affiliations:** 1 School of Medicine, Universidad de los Andes, Bogotá, Colombia; 2 Section of Endocrinology, Hospital Universitario Fundación Santa Fe de Bogotá, Bogotá, Colombia; 3 Division of Pulmonary, Critical Care and Sleep Medicine, Department of Medicine, Beth Israel Deaconess Medical Center and Harvard Medical School, Boston, Massachusetts, United States of America; 4 Molecular and Integrative Physiological Sciences Program, Department of Environmental Health, Harvard School of Public Health, Boston, Massachusetts, United States of America; Research Center Borstel, GERMANY

## Abstract

**Objectives:**

Our current understanding of hormone regulation in lung parenchyma is quite limited. We aimed to quantify a diverse array of biologically relevant protein mediators in alveolar lining fluid (ALF), compared to serum concentrations, and explore factors associated with protein compartmentalization on either side of the air-blood barrier.

**Research Design and Methods:**

Participants were 24 healthy adult non-smoker volunteers without respiratory symptoms or significant medical conditions, with normal lung exams and office spirometry. Cell-free bronchoalveolar lavage fluid and serum were analyzed for 24 proteins (including enteric and metabolic hormones, apolipoproteins, adipokines, and cytokines) using a highly sensitive multiplex ELISA. Measurements were normalized to ALF concentrations. The ALF:serum concentration ratios were examined in relation to measures of protein size, hydrophobicity, charge, and to participant clinical and spirometric values.

**Results:**

ALF measurements from 24 individuals detected 19 proteins, including adiponectin, adipsin, apoA-I, apoA-II, apoB, apoC-II, apoC-III, apoE, C-reactive protein, ghrelin, glucose-dependent insulinotropic peptide (GIP), glucagon-like peptide-1 (GLP-1), glucagon, insulin, leptin, monocyte chemoattractant protein-1, plasminogen activator inhibitor-1, resistin, and visfatin. C-peptide and serpin E1 were not detected in ALF for any individual, and IL-6, IL-10, and TNF-alpha were not detected in either ALF or serum for any individual. In general, ALF levels were similar or lower in concentration for most proteins compared to serum. However, ghrelin, resistin, insulin, visfatin and GLP-1 had ALF concentrations significantly higher compared to serum. Importantly, elevated ALF:serum ratios of ghrelin, visfatin and resistin correlated with protein net charge and isoelectric point, but not with molecular weight or hydrophobicity.

**Conclusions:**

Biologically relevant enteric and metabolic hormones, apolipoproteins, adipokines, and cytokines can be detected in the ALF of healthy individuals. For the proteins measured, charge may influence trafficking and compartmentalization to the alveolar airspace more than molecular weight or hydrophobicity. These data may have implications for homeostasis and drug delivery to the lung.

## Introduction

The alveolar lining fluid (ALF) is a highly specialized and tightly regulated liquid layer at the alveolar-epithelial cell interface that must fulfill the apparently diverse functions of preventing alveolar collapse while allowing quick and efficient gas exchange [1,2]. Even though the complex nature of ALF phospholipid and its role in controlling surface tension was described many decades ago, only recently has interest arisen about the protein components of the ALF and their biological role. The alveolar epithelium represents an appealing route for drug delivery for hormones such as insulin because of efficient absorption into the systemic circulation, paucity of drug-metabolizing enzymes and absence of a hepatic first-pass effect [1,2]. In order to exploit this attractive route for peptide therapeutics (which are usually administered by injection), it is important to have a thorough understanding of the ALF composition and its determinants. This includes the role of systemic hormones in the lungs, and their exchange with blood to and from the alveolar lining layer.

Many small molecules, such as urea, are thought to freely cross from circulation to the pulmonary interstitium and from there to the ALF, while larger ones would be restricted and thus should not be present in the ALF [3]. The threshold for size selectivity is approximately at the molecular weight of albumin (66 kDa) [4]. Nevertheless, there is very limited information on the actual concentration of proteins in human ALF, particularly those with ligand/endocrine activity. Most studies have focused on non-bioactive proteins [5], or on qualitatively identifying part of the repertoire of proteins in the ALF using proteomic tools [6–7]. Better knowledge of the protein components of ALF is particularly relevant now that many hormones and cytokines are being tested for pulmonary delivery, including insulin, human growth hormone, calcitonin, parathyroid hormone, interferon-beta, recombinant human granulocyte colony-stimulating factor, erythropoietin and glucagon-like peptide-1 [2,8]. We have previously studied the concentrations of insulin in ALF of patients with type 1 and 2 diabetes undergoing subcutaneous or inhaled insulin treatment [9], but very little is known about the concentrations of insulin and other hormones involved in metabolism or inflammation in healthy individuals.

The purpose of this study was to advance our understanding of alveolar and lung biology by investigating in the ALF of healthy adult individuals: [1]. The presence of biologically relevant proteins that may contribute to local cell regulation, [2]. Their relative concentrations (or compartmentalization) in the lung alveolar spaces compared to blood, and [3]. Potential mechanisms contributing to their relative accumulation or absence in ALF. For this purpose we selected a group of healthy non-smokers with normal spirometry, lung exam and with no respiratory symptoms, and measured in their bronchoalveolar lavage fluid proteins that are known to have an important role in metabolic regulation. We employed high-sensitivity techniques, in order to accurately estimate initial ALF concentrations before dilution. In addition, we explored essential protein characteristics that might be associated with their ALF concentrations. Since protein size and charge are the two fundamental properties of any protein, these were the factors we emphasized. We analyzed the concentrations of 24 different polypeptide hormones, cytokines and apolipoproteins. In addition, and as a secondary analysis, we explored correlation between clinical variables and the relative abundance of each protein in these two compartments.

For most of the select proteins examined, there is currently little or no evidence for their production by cells in the lungs, and their detection would suggest production elsewhere with subsequent crossing of the air-blood barrier to reach the alveolar lining layer.

## Methods

### Study participants

Healthy adult volunteers were recruited and provided written informed consent through a research protocol approved by the Beth Israel Deaconess Medical Center Committee on Clinical Investigations. All volunteers were non-smokers, without respiratory symptoms, and had a normal physical exam and spirometry. Current or former smokers, as well as individuals with a history of any chronic respiratory disease or current upper respiratory tract infection were excluded. Demographics were recorded for each volunteer, including age, gender, ethnicity, tobacco use, weight and height.

### Human bronchoalveolar lavage fluid and serum specimens

All procedures were performed by trained personnel in the bronchoscopy suite of the BIDMC West Procedures Center, a state-of-the-art facility with all necessary equipment to perform bronchoscopy safely, as previously described [10]. For all volunteers, following overnight fasting, airways were anesthetized with topical 1% lidocaine via nebulizer (oropharynx) and via bronchoscope (larynx and lower airways). With the volunteer in a semirecumbant position, the bronchoscope was placed in a wedged position via the oral route and bronchoalveolar lavage (BAL) was performed in a subsegment of the right middle lobe (RML) subsegment with instillation of 60 mL warm non-bacteriostatic normal saline, followed immediately by gentle aspiration (aspiration pressure 50–100 cm H2O, with the bronchoscope held firmly in place) into sterile collection traps. The BAL sequence was repeated a total of 4 times (total 240 ml normal saline instilled) and specimens were transported to the research laboratory on ice. Pooled BAL fluid was immediately centrifuged at 200g x 10 minutes at 4°C, and the cell-free supernatant removed, aliquoted and stored at -80°C until assayed. Generally, for the total 240 ml of instilled normal saline, the return of BAL fluid was 120–160 ml (representing 50–65% of the instilled normal saline volume). We set the minimum recovery at 50%, but did not have such a low recovery in any volunteer. For each bronchoscopy volunteer, serum samples were obtained by peripheral venipuncture using sterile technique during the same visit. The specimens were centrifuged and serum was aliquoted and stored at -80°C until assayed.

### Protein assays

BAL fluid (BALF) and serum were assayed for the following 24 hormones, apolipoproteins and inflammatory mediators using a multiplex Enzyme-Linked Immunosorbent Assay (ELISA) (Bio-Rad’s Bio-Plex Human Diabetes, Human Obesity and Human Apolipoprotein Panels, Eve Technologies, Calgary, Canada): adiponectin (lower limit of detection—LLD: 28.55 pg/mL), adipsin (LLD: 11.69 pg/mL), apoA-I (LLD: 1.28 ng/mL), apoA-II (LLD: 0.24 ng/mL), apoB (LLD: 0.99 ng/mL), apoC-II (LLD: 0.02 ng/mL), apoC-III (LLD: 0.05 ng/mL), apoE (LLD: 0.01 ng/mL), C-peptide (LLD: 0.99 pg/mL), C-reactive protein (LLD: 9.6 pg/mL), ghrelin (LLD: 1.98 pg/mL), GIP (LLD: 1.78 pg/mL), GLP-1 (LLD: 1.19 pg/mL), glucagon (LLD: 0.97 pg/mL), IL-10 (LLD: 0.93 pg/mL), IL-6 (LLD: 2.29 pg/mL), insulin (LLD: 1.78 pg/mL), leptin (LLD: 11.5 pg/mL), MCP-1 (LLD: 1.19 pg/mL), PAI-1 (LLD: 4.3 pg/mL), resistin (LLD: 1.54 pg/mL), serpin E1/PAI-1 (LLD: 4.9 pg/mL), TNF-alpha (LLD: 2.1 pg/mL), and visfatin (LLD: 83 pg/mL).

To perform multiplex ELISA on BALF, bovine serum albumin (BSA) was added to each sample as a carrier protein to optimize assay kinetics and to prevent BALF protein absorption to lab ware. BALF samples were assayed employing the following method: 20 microliters of BALF were spiked with 7 microliters of 2% BSA/phosphate buffered saline (PBS) to create a 0.5% BSA matrix in the samples. The BALF standard curve was also run in a 0.5% BSA/PBS matrix. The additional 1.35 dilution factor was used to calculate final concentrations. As the apolipoprotein panel included a carrier protein in the kit’s assay buffer, additional BSA was not added to these samples. Since the BAL procedure dilutes the ALF, all BALF data were normalized to urea measurements (BioAssay QuantiChrom colorimetric kit). We compared serum and BALF urea concentrations to estimate the dilution factor of analytes due to the BAL procedure for each participant [11]. Protein concentrations were expressed per ml of dilution-corrected estimates of ALF.

### Statistical methods

Comparison of median concentrations between serum and ALF were performed with Wilcoxon signed ranks test, a non-parametric statistical method. Correlations of serum and ALF concentrations, and of protein characteristics with ALF/serum concentration ratios, were determined by Spearman correlation coefficients. For the multiple correlations between ALF/serum ratios and pulmonary function tests and clinical variables, we used the Benjamini-Hochberg False Discovery Rate (FDR) adjustment method for multiple comparisons. The FDR procedures are statistical adjustment methods for multiple comparisons that keep the expected proportion of incorrectly rejected null hypotheses under a predetermined limit (alpha). This analysis has more power than family-wise error rates-based adjustment methods (like the Bonferroni method), which control the probability of making at least one incorrect rejection of a null hypothesis, but are more prone to type I error. However for this exploratory analysis, we considered higher power and a controlled false discovery rate to be preferable. For all analyses, we used a predetermined significance value of 5% (0.05).

## Results

Measures were taken to ensure reliability of the BALF measurements by following, the European Respiratory Society Guidelines recommendations [12], shown in S1 Table. A total of 24 healthy volunteers were included in the study (Table 1), including 7 women, with 16 (67%) white and 8 (33%) black. The mean age was 38.8 years and mean body mass index (BMI) was 26.3 kg/m^2^. All participants were non-smokers, and FEV1, FVC and FEV1/FVC were within normal limits for each subject.

**Table 1:** Characteristics of study participants.

n=	24
Female participants (%)	7 (29.1)
Age (years)	38.8 (12.1)
Weight (kg)	79.1 (20.8)
Height (m)	1.73 (0.09)
Body mass index (kg/m^2^)	26.3 (5.9)
Number of participants of black race	8 (33.3%)
Number of participants of white race	16 (66.7%)
Nonsmoker (%)	100
Homeostasis model assessment—insulin resistance (HOMA-IR) index	7.41 (18.4)
Forced vital capacity (FVC) (L)	4.55 (1.17)
Percent of expected FVC	106.2 (16)
Forced expiratory volume in the first second (FEV1) (L)	3.58 (0.94)
Percent of expected FEV1	108.9 (17.2)
FEV1 / FVC (%)	78.6 (4.25)
Percent of expected FEV1 / FVC (%)	108.9 (17.2)
Serum urea (mg/dL)	56.9 (17.2)
Bronchoalveolar lavage fluid urea (mg/dL)	2.50 (2.2)
Bronchoalveolar lavage fluid dilution factor	33.5 (25.4)

Data are means (SD) unless indicated otherwise.

### Measurements of hormones, cytokines and apolipoproteins in ALF and serum

ALF measurements from 24 individuals detected 19 proteins, including apolipoproteins (apoA-I, apoA-II, apoB, apoC-II, apoC-III and apoE), adipokines (leptin, adiponectin, adipsin, resistin and visfatin), metabolic and enteric hormones (ghrelin, glucose-dependent insulinotropic peptide [GIP], glucagon-like peptide-1 [GLP-1], glucagon and insulin) and cytokines (C-reactive protein, monocyte chemoattractant protein-1 and plasminogen activator inhibitor-1). C-peptide, serpin E1, IL-6, IL-10, and TNF-alpha were not detected in ALF for any individual. These five have all been omitted from subsequent analyses. Twenty-one proteins were detected in serum samples, whereas IL-6, IL-10 and TNF-alpha were not detected in serum samples for any individual.

### Correlation between ALF and serum concentrations

Table 2 lists the 24 proteins that were measurable, listed in the order of their concentration in ALF from the highest to the lowest. The third column from left to right gives the serum concentrations from these same subjects. In general, ALF levels were similar or lower in concentration for 14 of the measured proteins compared to serum for each individual. Table 3 compares each protein in both compartments and gives the ALF/serum ratio in the central column. Interestingly, for five of the measured proteins, ALF concentrations were markedly and significantly higher compared to serum: ghrelin, resistin, insulin, visfatin and GLP-1. Of the five analytes that were not detected in the ALF, IL-6, IL-10 and TNF-alpha were not detected in the serum from any individual. The high absolute levels of adipsin and adiponectin in the ALF only represented 1.6% and 0.2% of the serum concentrations, respectively.

**Table 2:** Concentrations of hormones and cytokines in serum and alveolar lining fluid.

Protein	Molecular weight (kDa)	Alveolar lining fluid concentration	Serum concentration
Resistin (pg/mL)	12.5	21,546 (10,008–45,292)	6,717 (5,895–9,797)
Ghrelin (pg/mL)	3.37	6,675 (2,024–13,802)	1,042 (662–1289)
Visfatin (pg/mL)	52	5,862 (0–15,507)	2,440 (1,671–4,265)
Insulin (pg/mL)	5.8	768 (511–1,254)	275 (209–551)
Leptin (pg/mL)	16	601 (466–1,097)	4,765 (1,579–9,188)
GLP-1 (pg/mL)	3.35	434 (227–877)	222 (205–262)
Glucagon (pg/mL)	3.49	317 (179–594)	302 (281–359)
MCP-1 (pg/mL)	11.0	143 (93–295)	216 (169–301)
Adipsin (ng/mL)	28	88 (57–241)	4,339 (3,513–4,662)
Adiponectin (ng/mL)	28	24 (16–47)	11,390 (7,995–17,228)
C-reactive protein (ng/mL)	115	10 (4.6–33.5)	888 (583–965)
ApoA-I (μg/mL)	28	0.61 (0.17–1.28)	1,195 (774–3,246)
PAi-1 (ng/mL)	43	0.22 (0.17–0.37)	46 (28–94)
ApoA-II (μg/mL)	11.2	0.11 (0.01–0.27)	321 (262–594)
ApoB (μg/mL)	512	0.07 (0–0.35)	99 (44–3,997)
ApoE (μg/mL)	34	0.06 (0.01–0.08)	54 (20–108)
ApoC-III (μg/mL)	8.7	0.03 (0.016–0.048)	181 (136–256)
ApoC-II (μg/mL)	11.2	0.003 (0.001–0.005)	78 (63–94)
GIP (pg/mL)	5.1	0 (0–141)*^a^	308 (237–401)

Proteins are ranked from the highest to the lowest concentration in ALF, after adjustment for dilution during the lavage procedure. Data are median (Q1, Q3).

^a^ Fourteen of the subjects had values of 0 and 10 had values ranging from 92.2 to 4,615.

**Table 3:** Concentrations of hormones and cytokines in serum and alveolar lining fluid.

Protein	Molecular weight (kDa)	ALF / serum concentration ratio (%)	P value
Ghrelin (pg/mL)	3.37	553 (135–1306)	<0.001
Resistin (pg/mL)	12.5	256 (124–810)	<0.001
Insulin (pg/mL)	5.8	242 (131–526)	0.019
Visfatin (pg/mL)	52	192 (0–547)	0.037
GLP-1 (pg/mL)	3.35	171 (105–370)	0.002
Glucagon (pg/mL)	3.49	102 (55–188)	0.71
MCP-1 (pg/mL)	11.0	90 (34–168)	0.84
Leptin (pg/mL)	16	14.2 (6.7–44)	<0.001
C-reactive protein (ng/mL)	115	2.13 (0.65–3.74)	<0.001
Adipsin (ng/mL)	28	1.65 (1.26–5.04)	<0.001
PAi-1 (ng/mL)	43	0.55 (0.19–0.88)	<0.001
Adiponectin (ng/mL)	28	0.22 (0.12–0.47)	<0.001
ApoE (μg/mL)	34	0.07 (0.04–0.22)	<0.001
ApoA-I (μg/mL)	28	0.04 (0.01–0.1)	<0.001
ApoA-II (μg/mL)	11.2	0.03 (0–0.11)	<0.001
ApoB (μg/mL)	512	0.02 (0–0.36)	<0.001
ApoC-III (μg/mL)	8.7	0.01 (0.01–0.03)	<0.001
GIP (pg/mL)	5.1	0 (0–42)	0.002
ApoC-II (μg/mL)	11.2	0 (0–0.01)	<0.001

Proteins are ranked from highest to lowest ALF / serum concentration, after adjustment for dilution during the lavage procedure. Data are median (Q1, Q3). P-value is for the difference between median concentrations in serum and median concentrations in ALF, from Wilcoxon′s signed-rank test. Proteins are sorted from the most to the least concentrated in ALF relative to serum.

The passive diffusion of proteins below a given size cutoff across the blood-ALF barrier should manifest as a positive correlation between serum and BALF concentrations; we found very little evidence of this. There was only one significant correlation between serum and BALF concentrations among the studied proteins (ApoE, Table 4). This suggests transport mechanisms in addition to purely passive diffusion based only on size selectivity; simple diffusion would have been evident from faster transport for smaller proteins.

**Table 4:** Correlation between alveolar lining fluid and serum concentrations of hormones and cytokines.

Protein	r =	Nominal p value	k	Benjamini-Hochberg criterion for rejection of Ho	Significant
ApoE	0.649	0.001	1	0,00263	Yes
C-reactive protein	0.542	0.009	2	0,00526	No
ApoA-II	-0.494	0.014	3	0,00789	No
PAi-1	-0.344	0.117	4	0,01053	No
Glucagon	0.309	0.162	5	0,01316	No
ApoB	0.314	0.178	6	0,01579	No
MCP-1	-0.223	0.294	7	0,01842	No
Resistin	0.217	0.31	8	0,02105	No
Adiponectin	0.212	0.32	9	0,02368	No
Visfatin	-0.209	0.327	10	0,02632	No
GIP	0.205	0.337	11	0,02895	No
ApoA-I	-0.204	0.338	12	0,03158	No
ApoC-II	0.11	0.626	13	0,03421	No
ApoC-III	0.10	0.658	14	0,03684	No
Adipsin	-0.063	0.768	15	0,03947	No
Leptin	-0.066	0.782	16	0,04211	No
Insulin	0.042	0.846	17	0,04474	No
GLP-1	0.033	0.881	18	0,04737	No
Ghrelin	0.008	0.971	19	0,05000	No

Data are Spearman correlation coefficients and their associated p-values from a hypothesis test evaluating whether the coefficients are different from zero. k: ordinal number of the comparison, from the most to the least significant according to the nominal p-value. The Benjamini-Hochberg FDR criterion for rejection of each p-value is shown, as well as whether or not the correlation is significant after FDR adjustment.

### Correlation of protein properties with ALF concentrations

#### Correlation with molecular weight

**Fig 1:**
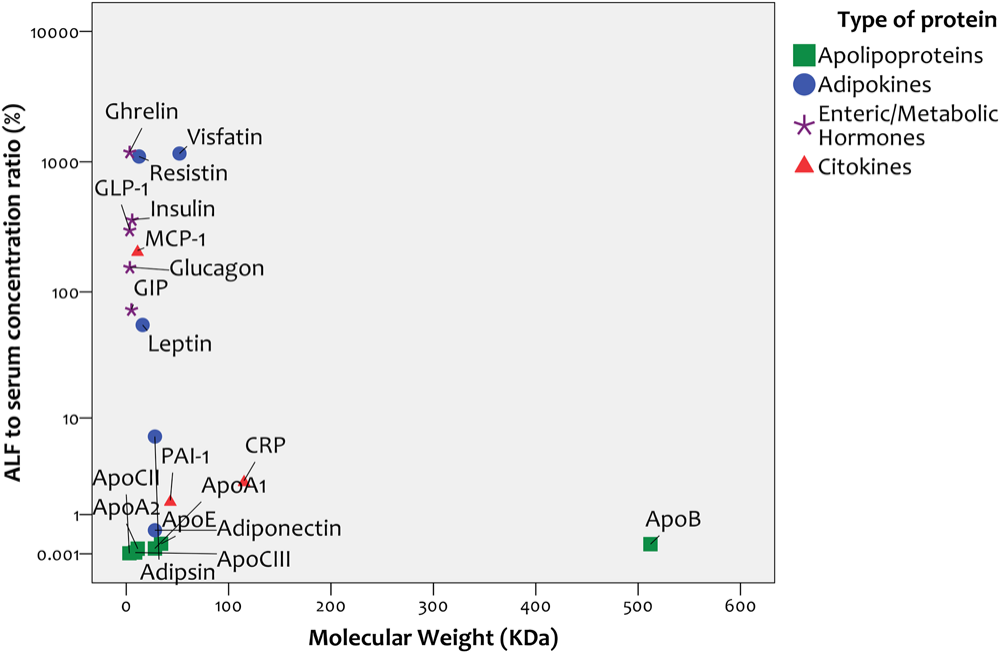
Correlation between protein molecular weight and alveolar lining fluid / serum ratio.

Despite including proteins with a broad range of molecular weights (range: 3–512 kDa), we found no correlation between the molecular weight of the measured proteins and their concentration in ALF relative to serum (Spearman r = -0.15, p = 0.53, Fig 1). When we analyzed this correlation separately for the 4 types of analytes studied (apolipoproteins, adipokines, hormones or cytokines), we found no correlation between molecular weight and concentration in ALF relative to serum within any of the four groups.

#### Correlation with hydrophobicity

**Fig 2:**
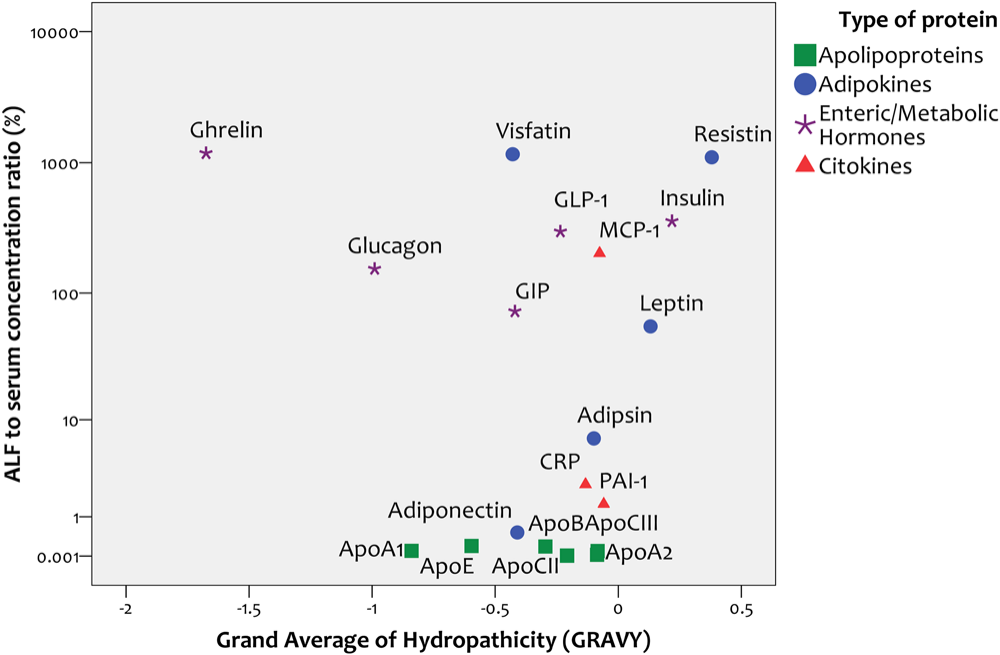
Correlation between protein hydrophobicity and alveolar lining fluid / serum ratio.

If proteins crossed the air-blood barrier primarily by dissolving in the membranes of cells that constitute the blood-ALF barrier, a positive correlation between the degree of protein hydrophobicity and concentration in ALF would be expected. We found no correlation between the overall degree of hydrophobicity of proteins, as reflected by their Grand Average of Hydropathicity (GRAVY) Index, and their concentration in ALF relative to serum (r = -0.24, p = 0.31, Fig 2). When we analyzed this correlation separately for the four types of analytes studied (apolipoproteins, adipokines, hormones or cytokines), we found no correlation between GRAVY and concentration in ALF relative to serum within any of the four groups.

#### Correlation with isoelectric point

**Fig 3:**
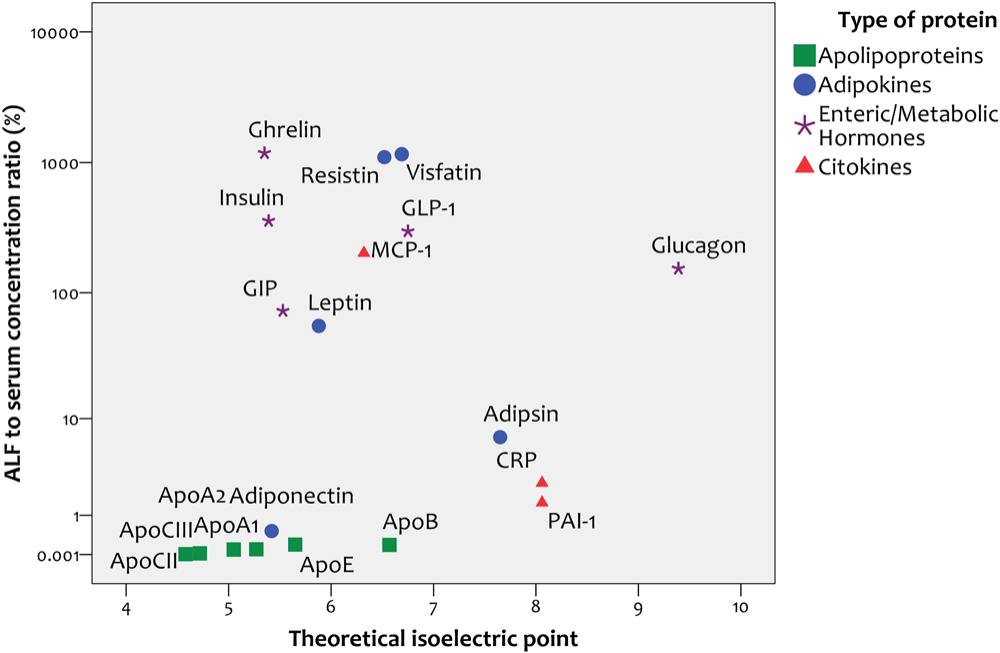
Correlation between protein isoelectric point and alveolar lining fluid / serum ratio.

Given that charge can be one of the most crucial and defining properties of polypeptides, we explored the correlation between the overall charge of the studied proteins, as reflected by their isoelectric point (pI), and their concentration in ALF relative to serum. We found a positive correlation between pI and degree of protein concentration in ALF relative to serum (r = 0.53, p = 0.015, Fig 3). Ghrelin, the protein most highly concentrated in ALF relative to serum, has a markedly basic theoretical pI of 9.62.

#### Correlation with net charge

**Fig 4:**
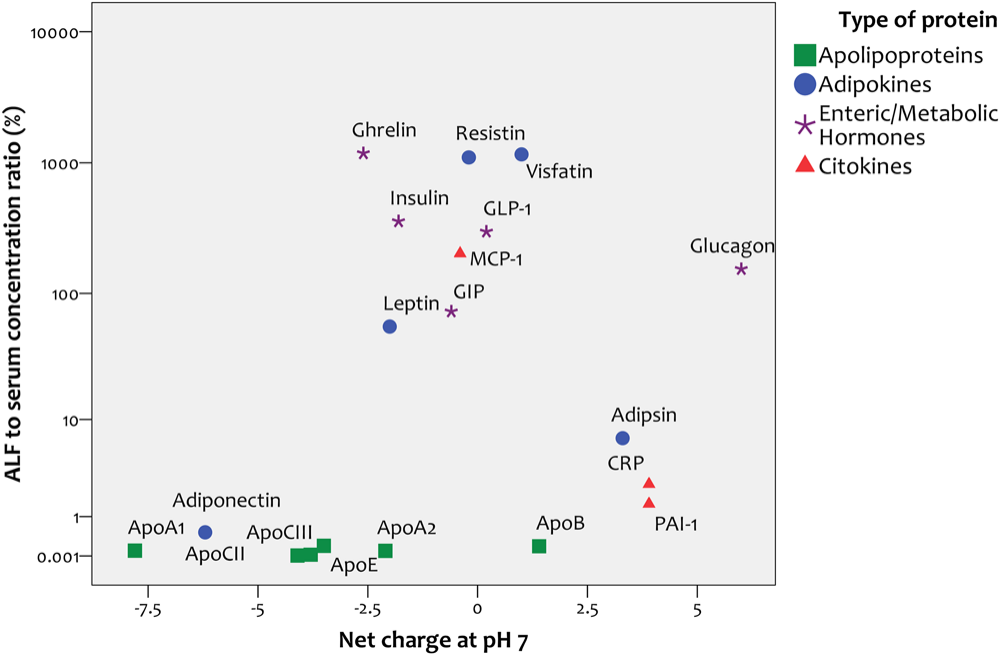
Correlation between protein charge at pH = 7 and alveolar lining fluid / serum ratio.

We found a positive correlation between the net charge of proteins at pH 7 (an approximation of physiological conditions) and their concentrations in ALF relative to serum (r = 0.58, p = 0.07, Fig 4).

Given that these results regarding protein charge and relative ALF abundance could be strongly influenced by three proteins highly concentrated in ALF that may act as outliers (ghrelin, visfatin and resistin), we tested these correlations excluding these 3 proteins. After excluding them, the correlation coefficient between ALF-to-serum ratios and theoretical isoelectric point was r = 0.55 (p = 0.028). Similarly, the correlation coefficient between ALF-to-serum ratios and protein charge at pH 7.0 was r = 0.54 (p = 0.031). Thus, significance was greater without these 3 proteins.

### Correlation between metabolic variables and ALF concentrations

After adjustment for multiple comparisons, we did not find significant correlations between fasting plasma glucose, BMI or HOMA-IR index and ALF-to-serum ratios of any the studied proteins (S2 Table).

### Correlation between pulmonary function tests and ALF concentrations

After adjustment for multiple comparisons, we did not find significant correlations between FEV1, FVC of the FEV1/FVC ratio and the ALF-to-serum ratio of any of the studied proteins (S3 and S4 Tables).

## Discussion

ALF measurements from 24 healthy non-smoking adults readily detected 19 proteins, including apolipoproteins, adipokines, enteric or metabolic hormones, and cytokines. Only C-peptide and serpin E1 were detected in serum but not in ALF. Interestingly, for five of the measured proteins, ALF concentrations were significantly higher compared to serum (ghrelin, resistin, insulin, visfatin and GLP-1). The detection of all these protein mediators, especially at higher levels than serum (i.e., compartmentalized), suggests a potential role in regulating cellular activity and maintaining lung homeostasis in the airways and alveolar airspaces.

To our knowledge, this is the first study to simultaneously measure a broad array of proteins in the ALF of healthy individuals, and to compare concentrations of these proteins in ALF to serum. We included molecules over a wide range of molecular sizes (3.4–512 kDa), but found that the relative distribution and concentrations of proteins in the ALF (compared to serum) did not correlate with molecular weight. This was unexpected and contrary to a compartimentalization mechanism based purely on limited passive diffusion.

We focused on proteins that play a major role in metabolic regulation throughout the body, many of which are larger than what has been considered the size limit to freely cross the air-blood barrier (about the size of albumin—66 KDa). The selected proteins were not known to be produced to a relevant degree by alveolar cells. We acknowledge that a high concentration in ALF may result from different mechanisms, and that it is not possible to quantify them using only the results of our study. These potential mechanisms include selective transport, diffusion in one direction but not the opposite, differential rates of proteolytic degradation, or charge/affinity. The finding of quite high concentrations of these biologically active proteins in ALF suggests a local role in alveolar biology, which may reflect specific transport mechanisms.

Proteomic studies of BALF in healthy controls or patients with diverse pulmonary occupational alterations did not find proteins with protease activity in the BALF of normal individuals [13]. Most alveolar proteases are concentrated in granules or intracellularly in alveolar macrophages, and released under conditions of inflammation [14]. In fact the same pattern (i.e. release mostly under inflammatory conditions) has been observed for natural pulmonary anti-proteases like SLPI and elafin [14]. Additionally, non-smokers tend to have very low activity of metalloproteinase in BALF [15]. Moreover, the risk of having protein components of BALF being degraded by proteases after recovery is minimized when the recovered BALF fluid is kept at temperatures between 0 and 4°C [16], as was the case in our study.

Lastly, increased proteolytic degradation could explain concentrations that are lower than those in serum to different degrees, but not concentrations markedly higher than those in serum, as we found for a few of the analyzed proteins. Thus, our results show that an important repertoire of proteins with ligand activity are present and in some cases concentrated in the ALF, and that size selectivity alone cannot be the main factor governing the protein composition of ALF under normal physiology.

The detection of multiple proteins in the ALF is consistent with reports by other investigators, although other reports measured fewer proteins, often only one. Depending on the question and purpose of their study, some of these proteins have been reported to be present in ALF. Our detection of certain proteins in the ALF confirms prior reports of specific mediators with ligand activity in the ALF, and supports a potential role for some of these proteins in normal alveolar biology. Apolipoprotein B100 was previously detected in proteomic studies of ALF [6]. Leptin and adiponectin, both detected in ALF in the current study, have cognate receptors expressed on pulmonary epithelial cells [17]. In addition to appetite regulation, leptin may function as a cytokine involved in the regulation of innate and adaptive immune responses [18]. Adiponectin binds to receptors in the respiratory epithelium and exerts a predominantly anti-inflammatory action [19]. Finally, metabolically active hormones like leptin [20] and adiponectin [21] may regulate the permeability of the capillary endothelium to other solutes. The absence of proinflammatory cytokines such as TNF-alpha and IL-6 in the ALF or serum of these study subjects is consistent with the absence of active disease or toxic exposures that might modulate ALF hormone levels. Inflammatory lung diseases elicit measurable cytokine concentrations in BALF [22,23].

The lack of correlation between ALF concentration and molecular weight challenges the common convention that molecular size is the predominant determinant of protein transport across biologic barriers like the air-blood barrier. Even though our data do not prove or rule out any particular transport mechanism for proteins into the ALF, it is also true that they do not support a transport mechanism based predominately on passive paracellular diffusion or bulk flow from serum to ALF. The paracellular route would be unlikely for large molecules based on the presence of type-1 pneumocyte tight and adherens junctions [24]. Alternatively, flow could occur through existing pores, as ultrastructural studies describe 4 nm intercellular spaces between type-1 pneumocytes [25] and a marked abundance of plasmalemmal vesicles (mean size about 70 nm) in the cytoplasm, representing an estimated 2% of total cell volume [26]. It has also been described that dog lungs have 8 nm alveolar epithelium pores [27]. Furthermore, studies in rat lung tissues report the rate of equilibration of small molecules and proteins of different sizes between serum and ALF occur at similar rates, something that supports a transcellular or vesicular transport mechanism [28]. However, in the current study, the lack of correlation between relative ALF concentrations (compared to serum) and protein hydrophobicity suggest that the mechanism involves other processes besides a passive transcellular pathway based purely on dissolution in plasma membranes. Results from the current study suggest a transport mechanism at least partially independent of molecular weight and hydrophobicity.

The mechanism for the compartmentalized distribution of select proteins (including ghrelin, resistin, insulin, visfatin and GLP-1) in part may be correlated with protein net charge and the isoelectric points of the proteins. The influence of net charge may reflect selective transport mechanisms that involve protein affinity for components of the alveolar interstitium or affinity for negatively charged components of the transcellular transport machinery. Prior studies show that moderately lipophilic compounds with a positive charge under physiological conditions (e.g., pentamidine or verapamil) have a greater affinity for lung tissue [29]. Still, it is possible that higher protein concentrations in the ALF may reflect local production by cells such as epithelial cells, which in turn serve or contribute to essential local biological function. For example, activated GLP-1 (GLP-1 7–36 amide) and a GLP-1 receptor agonist (exendin) induce surfactant phospholipid secretion by type 2 pneumocytes [30]. However, we are not aware of any evidence that GLP-1 is produced locally within the lungs. Higher ALF hormone levels may also reflect differences in local degradation by enzymes in the alveolar airspace, resulting in accumulation over time. However, since proteases and estearases tend to be concentrated either intracellularly in phagocytic cells or in the airway secretions, and are very scarce in the alveolar airspace [14], this mechanism would require selective degradation. While inflammation may result in increased protease release from degranulating neutrophils, the normal alveolar space is protease-poor [14]. This is consistent with the lack of degradation of insulin deposited in the lungs observed in clinical trials of inhaled insulin [9]. Accumulation of net positive charge proteins in the ALF may reflect greater affinity for ALF phospholipids (such as surfactant), thus limiting diffusion back into serum. Finally, increased ALF protein may reflect equilibrium between ALF and interstitium (rather than serum), as interstitial concentrations of hormones can be extremely high [31]. However, the number of interstitial cells adjacent to the alveoli is quite limited relative to epithelial and endothelial cells [3]. The observation that select hormones are compartmentalized to the ALF relative to serum suggests a potential important role in cellular and regulatory function. Defining the mechanism for the compartmentalization of select hormones represents an active area of investigation. To the best of our knowledge, and after repeating an extensive updated literature search, none of the studied proteins is produced locally by alveolar or other lung epithelial cells. In order to explore the possibility of local production of the proteins that were concentrated in ALF relative to serum (ghrelin, resistin, insulin, visfatin, GLP-1 and glucagon), we conducted a search in the European Bioinformatics Institute Human Gene Expression Atlas (http://www.ebi.ac.uk/gxa), to verify if their respective genes are expressed by lung cells (the map does not report on separate cell types within the lung). The resistin gene is expressed in the lung, albeit 8–10 fold less than in other tissues. The glucagon gene, which encodes both glucagon and GLP-1, is expressed in the lung at low levels, and the other genes are not expressed in the lungs.

We did not find significant correlations between participants′ characteristics of metabolic disturbances (plasma glucose, BMI, HOMA-IR index), and the relative concentration of metabolic hormones in ALF. The same was true for pulmonary function tests. Correlation of ALF hormone levels in persons with abnormal spirometry would be of great interest for future studies.

Even though this study opens new research avenues focused on the potential sources and transport of the proteins we studied, it is limited by a small sample size, and by the very strict selection criteria that were applied to potential volunteers. We do not know the extent to which these findings would be replicated in a different sample of healthy individuals, or in patients with significant systemic or pulmonary comorbidities. We must also recognize that the dilution factor generated from the measurement of urea in the BALF is subject to limitations. There may be some transit of urea from the circulation to BALF during the lavage procedure [32,33]. Nevertheless, the extent of this diffusion is dependent on the dwell time of the wash fluid during the BAL procedure, which in our case was less than one minute. Also, transit of urea from circulation to BALF would lead to a smaller correction factor, and an underestimation of the concentration of solutes in ALF, but not to the high concentrations we found for very specific proteins. We must also consider that the number of data points used to compute correlations between protein properties and their ALF to serum ratio was small (19 proteins), and even though we employed a correlation coefficient that is more robust to small sample sizes (Spearman′s), it is likely that analyses of a larger number of proteins might find some correlation between molecular weight or hydrophobicity and relative ALF abundance in normal subjects.

Lastly, an inevitable limitation is the fact that the biology of these ligands within the respiratory system has not been studied very thoroughly. From our information we are not able to infer whether each of these proteins is being transported from the circulatory system, from the pulmonary interstitium, or locally produced by alveolar cells. Especially, their biologic roles in lung biology, if any, need to be characterized.

In conclusion, our study demonstrates that critical and biologically relevant enteric and metabolic hormones, apolipoproteins, adipokines, and cytokines are present in the alveolar lining fluid of healthy individuals. Protein concentrations may be influenced by BMI, the presence of insulin resistance, and may influence lung mechanics. These ALF proteins may participate in regulating inflammation and metabolism in parenchymal lung cells, and may influence immune cells in the alveolar airspace such as resident macrophages. Furthermore, physical properties of proteins such as charge (but not molecular weight or hydrophobicity) may preferentially influence hormone trafficking and compartmentalization to the alveolar airspace. This has potential implications for homeostasis and for aerosolized drug delivery to and through the lungs if the delivered agents modify protein concentrations in the ALF.

## Supporting Information

S1 File Study Dataset. (CSV)

S1 Table Compliance with European respiratory Society Guidelines for measurement of acellular components of BAL in the study (12) (DOCX)

S2 Table Correlation between body mass index, Homeostasis Model Assessment—Insulin Resistance (HOMA-IR) index, and alveolar lining fluid / serum ratio of hormones and cytokines. Data are Spearman correlation coefficients and their associated p-values from a hypothesis test evaluating whether the coefficients are different from zero. BH criterion: Benjamini-Hochberg FDR criterion for rejection of each p-value. BMI: Body-mass index.
(DOCX)

S3 Table Correlation between pulmonary function tests (percent of expected forced vital capacity and forced expiratory volume in the first second) and alveolar lining fluid / serum ratio of hormones and cytokines (n = 13). Data are Spearman correlation coefficients and their associated p-values from a hypothesis test evaluating whether the coefficients are different from zero. BH criterion: Benjamini-Hochberg FDR criterion for rejection of each p-value. Nom. p value: Nominal p-value, Sign: Significant or nor after FDR adjustment.
(DOCX)

S4 Table Correlation between (forced expiratory volume in the first second/forced vital capacity ratio—FEV1/FVC) and alveolar lining fluid / serum ratio of hormones and cytokines (n = 13). Data are Spearman correlation coefficients and their associated p-values from a hypothesis test evaluating whether the coefficients are different from zero. BH criterion: Benjamini-Hochberg FDR criterion for rejection of each p-value. Nom. p value: Nominal p-value, Sign: Significant or nor after FDR adjustment.
(DOCX)
