# Supplementary material for: Metabolic Hormones, Apolipoproteins, Adipokines, and Cytokines in the Alveolar Lining Fluid of Healthy Adults: Compartmentalization and Physiological Correlates
Source: PLoS One. 2015 Apr 7;10(4):e0123344. doi: 10.1371/journal.pone.0123344 (PMC4388476; doi:10.1371/journal.pone.0123344)
Supplement: S1 Table — (DOCX) [file pone.0123344.s002.docx]

**Supplemental Table 1.** Compliance with European respiratory Society Guidelines for measurement of acellular components of BAL in the study (12)

| **Source of variability** | **ERS Recommendation** | **Situation in the study** |
| --- | --- | --- |
| Disease process itself | Specify underlying disease | All participants were apparently healthy, had no respiratory symptoms, and normal spirometry |
| Smoking | Specify whether non-, ex- or current smoker | We excluded smokers |
| Drug treatment | Specify whether on antiinflammatory or other drugs | Participants were not taking any medication |
| Associated diseases | Specify any relevant associated diseases, *e.g.* asthma | All participants were apparently healthy, had no respiratory symptoms, and normal spirometry |
| Dwell time before aspiration of fluid | Keep to a minimum, and specify dwell time if prolonged | Once appropriately anesthetized with topical lidocaine, a 60 ml aliquot of normal saline (room temperature) was instilled through the bronchoscope into the right middle lobe subsegment, and immediately suctioned (to minimize dwell time) |
| Suction pressure during aspiration | Keep to a minimum (25–100 mmHg) | Constant suction pressure 50-100 mmHg with the bronchoscope held firmly in place |
| Physician doing lavage procedure | Specify | Henry Koziel |
| Contamination from airways | Determine epithelial cell counts standardly | In all BAL fluid cell counts, epithelial cells accounted for <0.1 % of total BAL cells. Airway mucus contamination was minimal. |
| The handling of lavage fluid (*e.g.* filtered, unfiltered, dithiothreitol, concentrated, other) | State which technique | Pooled BAL fluid was immediately centrifuged at 200g x 10 minutes at 4^o^C, and the cell-free supernatant removed, aliquoted and stored at -80^o^C until assayed. |
| Volume of instilled fluid | Use ~100 mL in adults and report volume standardly instilled (European Task Force recommended 200–240 mL^-1^) | Instillation was repeated in the same location for an additional 3 aliquots of 60 ml normal saline, and the return BAL fluid pooled into sterile traps. |
| Number of aliquots instilled | Specify and standardize (European Task Force recommended four) | Instillation was repeated in the same location for an additional 3 aliquots of 60 ml normal saline (total 4 aliquots), and the return BAL fluid pooled into sterile traps. |
| Position of patient | Specify | Volunteers were in semi-recumbent position |
| Area which is lavaged (one or more lobes, right middle lobe, right lower lobe, other?) | Specify | Right middle lobe subsegment |
| Variability of return of lavage fluid | Report volume and percentage of fluid recovered; establish criteria for minimum recovery | Generally, for the total 240 ml of instilled normal saline, the return of BAL fluid was 120-160 ml (representing 50-65% of the instilled normal saline volume). We set the minimum recovery at 50%, but did not have such a low recovery in any volunteer. |
| Reporting measurements of acellular components in BAL | Report values per mL of BAL fluid recovered (as well as any other special approaches) | We report per mL of ALF, using a correction factor derived from an endogenous marker (urea) |
| Sample handling and storage | Refer to recommendations in specific sections of this task force report | Pooled BAL fluid was immediately centrifuged at 200g x 10 minutes at 4^o^C, and the cell-free supernatant removed, aliquoted and stored at -80^o^C until assayed. |
| Assay procedures and assay controls | Refer to recommendations in specific sections of this task force report | Explained in detail under “protein assays”. |
