# Supplementary material for: Metabolic Hormones, Apolipoproteins, Adipokines, and Cytokines in the Alveolar Lining Fluid of Healthy Adults: Compartmentalization and Physiological Correlates
Source: PLoS One. 2015 Apr 7;10(4):e0123344. doi: 10.1371/journal.pone.0123344 (PMC4388476; doi:10.1371/journal.pone.0123344)
Supplement: S2 Table — Data are Spearman correlation coefficients and their associated p-values from a hypothesis test evaluating whether the coefficients are different from zero. BH criterion: Benjamini-Hochberg FDR criterion for rejection of each p-value. BMI: Body-mass index. (DOCX) [file pone.0123344.s003.docx]

**Supplemental Table 2. Correlation between body mass index, Homeostasis Model Assessment – Insulin Resistance (HOMA-IR) index, and alveolar lining fluid / serum ratio of hormones and cytokines.** Data are Spearman correlation coefficients and their associated p-values from a hypothesis test evaluating whether the coefficients are different from zero. BH criterion: Benjamini-Hochberg FDR criterion for rejection of each p-value. BMI: Body-mass index.

|  | **BMI** | | | | **HOMA-IR** | | | |
| --- | --- | --- | --- | --- | --- | --- | --- | --- |
| **BH criterion** | **Protein** | **r=** | **Nominal p value** | **Significant** | **Protein** | **r=** | **Nominal p value** | **Significant** |
| 0.0026 | Ghrelin | 0.551 | 0.006 | No | C-reactive protein | 0.46 | 0.031 | No |
| 0.0053 | Adipsin | 0.516 | 0.01 | No | Insulin | -0.296 | 0.16 | No |
| 0.0079 | Visfatin | 0.518 | 0.01 | No | ApoE | 0.292 | 0.167 | No |
| 0.0105 | Glucagon | 0.419 | 0.052 | No | ApoB | -0.224 | 0.343 | No |
| 0.0132 | GLP-1 | 0.401 | 0.058 | No | Leptin | -0.179 | 0.45 | No |
| 0.0158 | ApoE | 0.289 | 0.17 | No | Adipsin | -0.161 | 0.451 | No |
| 0.0184 | ApoA-I | 0.264 | 0.212 | No | ApoA-I | -0.153 | 0.475 | No |
| 0.0211 | MCP-1 | 0.248 | 0.243 | No | ApoA-II | -0.147 | 0.492 | No |
| 0.0237 | Resistin | 0.207 | 0.332 | No | PAi-1 | -0.111 | 0.622 | No |
| 0.0263 | Adiponectin | 0.182 | 0.394 | No | Glucagon | -0.097 | 0.669 | No |
| 0.0289 | ApoB | -0.19 | 0.422 | No | Visfatin | -0.089 | 0.678 | No |
| 0.0316 | Insulin | 0.159 | 0.457 | No | Ghrelin | -0.086 | 0.697 | No |
| 0.0342 | PAi-1 | 0.135 | 0.549 | No | GIP | -0.077 | 0.721 | No |
| 0.0368 | GIP | 0.103 | 0.631 | No | Adiponectin | -0.076 | 0.724 | No |
| 0.0395 | ApoC-II | -0.086 | 0.703 | No | GLP-1 | -0.077 | 0.727 | No |
| 0.0421 | Leptin | -0.087 | 0.714 | No | Resistin | -0.073 | 0.733 | No |
| 0.0447 | ApoA-II | -0.068 | 0.753 | No | MCP-1 | -0.065 | 0.764 | No |
| 0.0474 | ApoC-III | -0.049 | 0.827 | No | ApoC-II | -0.044 | 0.844 | No |
| 0.0500 | C-reactive protein | -0.004 | 0.985 | No | ApoC-III | 0.013 | 0.955 | No |
