# Supplementary material for: Metabolic Hormones, Apolipoproteins, Adipokines, and Cytokines in the Alveolar Lining Fluid of Healthy Adults: Compartmentalization and Physiological Correlates
Source: PLoS One. 2015 Apr 7;10(4):e0123344. doi: 10.1371/journal.pone.0123344 (PMC4388476; doi:10.1371/journal.pone.0123344)
Supplement: S3 Table — Data are Spearman correlation coefficients and their associated p-values from a hypothesis test evaluating whether the coefficients are different from zero. BH criterion: Benjamini-Hochberg FDR criterion for rejection of each p-value. Nom. p value: Nominal p-value, Sign: Significant or nor after FDR adjustment. (DOCX) [file pone.0123344.s004.docx]

**Supplemental Table 3.** **Correlation between pulmonary function tests (percent of expected forced vital capacity and forced expiratory volume in the first second) and alveolar lining fluid / serum ratio of hormones and cytokines (n=13).** Data are Spearman correlation coefficients and their associated p-values from a hypothesis test evaluating whether the coefficients are different from zero. BH criterion: Benjamini-Hochberg FDR criterion for rejection of each p-value. Nom. p value: Nominal p-value, Sign: Significant or nor after FDR adjustment.

|  | **% of expected FVC** | | | | **% of expected FEV1** | | | |
| --- | --- | --- | --- | --- | --- | --- | --- | --- |
| **BH criterion** | **Protein** | **r=** | **Nom.p value** | **Sign.** | **Protein** | **r=** | **Nominal p value** | **Sign.** |
| 0,0026 | Adiponectin | 0,660 | 0,014 | No | ApoA-II | 0,687 | 0,010 | No |
| 0,0053 | ApoA-I | 0,584 | 0,036 | No | ApoA-I | 0,663 | 0,014 | No |
| 0,0079 | ApoA-II | 0,542 | 0,056 | No | Adiponectin | 0,582 | 0,037 | No |
| 0,0105 | ApoC-II | 0,426 | 0,146 | No | ApoE | 0,516 | 0,071 | No |
| 0,0132 | MCP-1 | 0,404 | 0,171 | No | ApoC-II | 0,511 | 0,074 | No |
| 0,0158 | ApoC-III | 0,396 | 0,180 | No | GIP | 0,503 | 0,080 | No |
| 0,0184 | ApoE | 0,391 | 0,187 | No | Leptin | 0,539 | 0,108 | No |
| 0,0211 | CRP | 0,330 | 0,271 | No | ApoC-III | 0,451 | 0,122 | No |
| 0,0237 | GIP | 0,306 | 0,310 | No | Ghrelin | 0,407 | 0,168 | No |
| 0,0263 | GLP-1 | 0,261 | 0,412 | No | MCP-1 | 0,390 | 0,188 | No |
| 0,0289 | Leptin | 0,285 | 0,425 | No | ApoB | 0,395 | 0,230 | No |
| 0,0316 | PAi-1 | 0,203 | 0,527 | No | GLP-1 | 0,326 | 0,301 | No |
| 0,0342 | Ghrelin | 0,193 | 0,528 | No | Insulin | 0,297 | 0,325 | No |
| 0,0368 | Insulin | 0,184 | 0,547 | No | PAi-1 | 0,294 | 0,354 | No |
| 0,0395 | Resistin | -0,118 | 0,700 | No | Glucagon | 0,245 | 0,443 | No |
| 0,0421 | ApoB | 0,128 | 0,707 | No | Adipsin | 0,154 | 0,616 | No |
| 0,0447 | Glucagon | 0,105 | 0,745 | No | CRP | 0,154 | 0,616 | No |
| 0,0474 | Visfatin | 0,096 | 0,754 | No | Visfatin | 0,128 | 0,677 | No |
| 0,0500 | Adipsin | 0,063 | 0,837 | No | Resistin | -0,027 | 0,929 | No |
