# Supplementary material for: Metabolic Hormones, Apolipoproteins, Adipokines, and Cytokines in the Alveolar Lining Fluid of Healthy Adults: Compartmentalization and Physiological Correlates
Source: PLoS One. 2015 Apr 7;10(4):e0123344. doi: 10.1371/journal.pone.0123344 (PMC4388476; doi:10.1371/journal.pone.0123344)
Supplement: S4 Table — Data are Spearman correlation coefficients and their associated p-values from a hypothesis test evaluating whether the coefficients are different from zero. BH criterion: Benjamini-Hochberg FDR criterion for rejection of each p-value. Nom. p value: Nominal p-value, Sign: Significant or nor after FDR adjustment. (DOCX) [file pone.0123344.s005.docx]

**Supplemental Table 4.** **Correlation between (forced expiratory volume in the first second/forced vital capacity ratio - FEV1/FVC) and alveolar lining fluid / serum ratio of hormones and cytokines (n=13).** Data are Spearman correlation coefficients and their associated p-values from a hypothesis test evaluating whether the coefficients are different from zero. BH criterion: Benjamini-Hochberg FDR criterion for rejection of each p-value. Nom. p value: Nominal p-value, Sign: Significant or nor after FDR adjustment.

| **BH criterion** | **Protein** | **r=** | **Nom. p value** | **Sign.** |
| --- | --- | --- | --- | --- |
| 0,0026 | Insulin | 0,000 | 1,000 | No |
| 0,0053 | CRP | -0,487 | 0,091 | No |
| 0,0079 | ApoB | 0,508 | 0,111 | No |
| 0,0105 | Leptin | -0,502 | 0,139 | No |
| 0,0132 | ApoC-III | -0,276 | 0,362 | No |
| 0,0158 | Resistin | 0,275 | 0,362 | No |
| 0,0184 | MCP-1 | 0,266 | 0,380 | No |
| 0,0211 | Ghrelin | 0,256 | 0,399 | No |
| 0,0237 | GLP-1 | 0,213 | 0,506 | No |
| 0,0263 | PAi-1 | 0,212 | 0,508 | No |
| 0,0289 | GIP | 0,183 | 0,551 | No |
| 0,0316 | Visfatin | 0,177 | 0,564 | No |
| 0,0342 | ApoA-I | -0,141 | 0,647 | No |
| 0,0368 | Adiponectin | -0,102 | 0,740 | No |
| 0,0395 | Glucagon | 0,095 | 0,769 | No |
| 0,0421 | ApoC-II | -0,054 | 0,860 | No |
| 0,0447 | ApoA-II | -0,022 | 0,943 | No |
| 0,0474 | ApoE | -0,011 | 0,973 | No |
| 0,0500 | Adipsin | 0,007 | 0,983 | No |
